# Contributions of digital social research to develop Telemedicine in Calabria (Southern Italy): identification of inequalities in post-COVID-19

**DOI:** 10.3389/fsoc.2023.1141750

**Published:** 2023-05-09

**Authors:** Luciana Taddei, Francesco Mendicino, Teresa Grande, Antonella Mulé, Roberto Micozzi, Ercole Giap Parini

**Affiliations:** ^1^Department of Political and Social Sciences, University of Calabria, Cosenza, Italy; ^2^U.O.C. Hematology, Annunziata Hospital, Cosenza, Italy; ^3^Independent Consultant, London, United Kingdom

**Keywords:** Telemedicine, digital social research, COVID-19, inequalities, Calabria

## Abstract

The paper discusses the role that sociology and digital social research methods could play in developing E-health and Telemedicine, specifically after the COVID-19 pandemic, and the possibility of dealing with new pandemics. In this article, we will reflect on an interdisciplinary research pilot project carried out by a team of sociologists, medical doctors, and software engineers at The University of Calabria (Italy), to give a proof of concept of the importance to develop Telemedicine through the contribution of digital social research. We apply a web and app survey to administrate a structured questionnaire to a self-selected sample of the University Community. Digital social research has highlighted socioeconomic and cultural gaps that affect the perception of Telemedicine in the University Community. In particular, gender, age, educational, and professional levels influence medical choices and behaviors during Covid-19. There is often an unconscious involvement in Telemedicine (people use it but don't know it is Telemedicine), and an optimistic perception grows with age, education, professional, and income levels; equally important are the comprehension of digital texts and the effective use of Telemedicine. Limited penetration of technological advances must be addressed primarily by overcoming sociocultural and economic barriers and developing knowledge and understanding of digital environments. The key findings of this study could help direct public and educational policies to reduce existing gaps and promote Telemedicine in Calabria.

## 1. Introduction

The pandemic highlighted how new technologies made it possible to solve problems that would have otherwise remained unsolved. Digital social research, applied in an interdisciplinary project, proved an indispensable tool for carrying out research when it would not have been possible to apply any other techniques and obtain important findings to develop Telemedicine in the territorial area of Calabria. The following will frame the object and the context of research, the role of digital research methods in identifying inequalities, and finally, the developed project.

### 1.1. E-Health and Telemedicine in Italy and Calabria

E-Health is defined as the application of information and communication technologies to health and care (World Health Organization, 2023). As in other areas (e-business, e-learning, e-commerce, etc.,), the prefix “e” stands for the application of “electronic” technologies, in this case specifically applied to health care. It is a relatively recent term, arising around 1999 in industry and marketing before academia (Eysenbach, 2001).

In 2005, the World Health Assembly defined e-health as ≪the cost-effective and secure use of information and communications technology (ICT) in support of health and health-related fields, including healthcare services, health surveillance, health literature, and health education, knowledge, and research≫ embracing a variety of application forms. Indeed, several branches of medicine incorporate ICT over time, each at different rates and proportions (Reichertz, 2006). Still, most importantly, its technical development comes with a change of perspective, which improves medical science in its local, regional, and global applications (Eysenbach, 2001).

E-Health opens unlimited possibilities for interaction between the health service provider and user (World Health Organization, 2016), between one health institution and another, and between users themselves in a peer-to-peer scenario (Eysenbach, 2001); in this sense, clearly defining its use is still an open challenge in the context of medical-scientific progress (Souza Filho and Monteiro, 2022).

The research project presented in this paper focuses on a specific branch of e-Health, namely Telemedicine. Telemedicine aims to provide proper remote health services (Sood et al., 2007) and has become prominent with the COVID-19 crisis (Yasmeen et al., 2021).

As defined by the (Italian Ministry of Health, 2012, p. 10): ≪Telemedicine is a way of delivery of health care services, through the use of innovative technologies, in particular Information and Communication Technologies (ICT), in situations where the health professional and the patient (or two professionals) are not in the same location≫. It must ensure ≪the secure transmission of medical information and data in the form of text, sound, images or other forms necessary for the prevention, diagnosis, treatment and subsequent monitoring of patients≫ (Italian Ministry of Health, 2012, p. 10). It is specified that it does not replace traditional medicine. Still, it aims to “improve efficacy, efficiency and appropriateness” of health care delivery by focusing on the citizen. Therefore, Telemedicine's objectives can be secondary prevention, diagnosis, treatment, rehabilitation, or monitoring. It can be divided into three main macro-areas: specialized Telemedicine (declined in televisita, teleconsultation, or health telecooperation), tele-health (mainly dedicated to chronic diseases), tele-assistance (reserved for the elderly, frail, or people with disabilities).

In connection with various national and international studies on the topic (see the collection made by Sood et al., 2007), the chief advantages of Telemedicine are the ability to address territorial inhomogeneity and the lack of infrastructure or social healthcare providers (Sood et al., 2007) all issues that closely affect the Italian context (Giarelli and Giovannetti, 2019; Benvenga, 2021).

In Italy, the National Guidelines on Telemedicine approved in 2012 aim to provide a unified and organic framework (Italian Ministry of Health, 2012). The report highlights how Telemedicine experiences in Italy are experimental, limited, and fragmented. It proposes models for its integration into the Italian National Health Service (NHS), considering technical and ethical aspects.

In 2019, the Italian government built a national map of active Telemedicine instances, identifying 282 initiatives mainly concentrated in a few regions in Northern and Central Italy. In Calabria, one of the most disadvantaged regions in the South, the report identified only three instances specifically concerning the remote control of Pacemakers, Defibrillators, Loop Recorders, as well as radiological and cardiological tele-referral (Italian Ministry of Health, 2012).

As Omboni (2020) points out, despite these valid foundations, Italy showed in COVID-19 a severe lag in digital support to medical care, and interest in Telemedicine only increased exponentially when an urgent need emerged.

Today, the willingness to continue along this path is evident in Mission 6—Health—of the Recovery and Resilience Plan, whose ≪objective is to bridge the gap between territorial disparities and offer greater integration between regional health services and national platforms, through innovative solutions. ≫It is stressed that ≪the development of Telemedicine is among the interventions to make the *Home* the first place of care≫ (Italian Ministry of Health, 2022).

The focus on domiciliary care was a defining feature of the pandemic period; it was only with the crisis triggered by the emergency that some of the structural problems of our National Health Service became evident (Giarelli, 2021). In particular, the gradual defunding, corporatization, and regionalization of the NHS in recent decades (Giarelli, 2017) has not put local healthcare providers in a position to effectively limit the spread of COVID-19 (Giarelli and Vicarelli, 2020).

In addition to structural issues, another obstacle to the development of technological innovations is Italy's low digitalization index (20th out of 27 EU countries) (European Commission, 2022, p. 3). The Digitization of Economy and Society 2021 Index (DESI) shows that, although Italian public services have recently invested in digital innovation, they remain below the European average. The same document also highlights the lack of digital skills among the Italian population and slow and not widespread internet connections, ≪although Italy has made progress in terms of both coverage and deployment of connectivity networks. The pace of fiber deployment has slowed between 2019 and 2020, and further efforts are needed to increase the coverage of ultra-high-capacity and 5G networks and encourage their deployment≫ (European Commission, 2022, p. 3).

A 2013 Di Carlo and Santarelli study highlighted how digital medicine development follows the country's infrastructural, technological, and economic development, confirming the gap between Northern Italy and the rest of the country. In particular, Calabria and Campania had the lowest innovation levels (Di Carlo and Santarelli, 2013).

The University of Calabria is, however, an excellence hub (Bianchi, 2011; Aiello et al., 2012), where it is possible to develop interdisciplinary projects, useful to manage the current emergency and to clear the way for social and health innovation.

### 1.2. Contributions of digital social research: identification of inequalities

The University of Calabria is an excellent place (Aiello et al., 2012) to develop an interdisciplinary project in which medicine, technology, and social science interact. The University of Calabria has a highly qualified University Health Center and Center of Information and Communication Technology, and highly qualified Departments of (1) Pharmacy, Health, and Nutrition Sciences, (2) Computer, Modeling, Electronic, and Systems Engineering, and (3) Political and Social Sciences.

Social sciences, in this case, must individuate a method that can be integrated with Telemedicine and can be used in the pandemic period, avoiding physical contact. Of course, all digital methods have these characteristics. Still, considering the sample population, it is also necessary to choose a technique able to detect a large amount of data on a large population, allowing us to make good descriptions and inferences about the university community (Langbecker et al., 2017).

Digital social research ≪tends to be used to refer to conducting “e-research” using digitalized data sets […]. The focus, therefore, is on the collection and use of data and the tools to analyze these data≫ (Lupton, 2012, p. 7), as we will see (par. 2.1), our technological solution can collect and give a first description of data.

Digital social research increased in the last 10 years (Veltri, 2021). It includes multiple quantitative and qualitative methods (Lupton, 2018, p. 42). Some come from social research tradition and are applied to the digital (e.g., content analysis, ethnography, surveys), while others are natively digital methods. Also, the kind of data that they analyze is different. Mainly we divide it into digitized and natively digital data (Rogers, 2016, p. 40): the former are analogic objects transformed into digital data, and the latter are data created directly in digital form. Another important distinction is between unobtrusive data collection methods (web scraping, social media mining) and obtrusive methods (qualitative methods, web surveys, experiments), which raise different questions, limitations, and reflections (Veltri, 2021).

This paper focuses on web and app survey, a traditional obtrusive method transformed into a digital one. In this case, we use it to reach a population potentially unattainable at the time of COVID-19, to reduce costs, and to speed up detection (Veltri, 2021, pp. 72–73), also considering the broader framework in which the research was developed (see par. 1.3. The project). In particular, we offered a Telemedicine service, so it was necessary to flank a social research method well integrated with it. It could potentially arrive at all populations investigated (Veltri, 2021, p. 85–89).

Many studies underline the advantages of Telemedicine (Finkelstein et al., 2006; Coulter, 2012; Weinstein et al., 2014). However, some studies demonstrate that some initiatives fail (Elwyn et al., 2012; Zanaboni and Wootton, 2012; Armfield et al., 2014). Lupton and Masley (2017), in their recent review, underline how ≪the social contexts and ethical implications are often not fully considered≫ and how ≪researchers have rarely addressed these issues in great depth in their evaluations of Telemedicine≫. In a context like the South of Italy, as we have already seen, it will be essential to take into consideration sociological variables to develop Telemedicine. But why?

As reported in the Glossary of Health Inequalities (Kawachi et al., 2002, p. 647): ≪*Health inequality* is the generic term used to designate differences, variations, and disparities in the health achievements of individuals and groups. […] Health inequality is a descriptive term that need not imply moral judgment≫, it is a condition that does not depend on individuals. In our definition, it leads with opportunities and not with outcomes (Scambler, 2011).

The health inequalities that have received more attention are gender, ethnic and spatial relations (Graham, 2009; Annendale, 2010; Bradby and Nazroo, 2010), but also the social position (Link and Phelan, 1995), behavior (Bartley, 2003), material (Gray, 1982) and psychosocial factors (Wilkinson, 1996).

At the origin, Telemedicine was presented as a way to overcome inequalities (Williams, 2003), but it cannot be made if there remain inequalities in access to technology (Cotton and Gupta, 2004; DiMaggio et al., 2004). Recent research (Heponiemi et al., 2020) underlines how access to online services, ICT skills, and extent of use is associated with the perception of Telemedicine benefits and the importance of having good relationships in everyday life.

Overall, in Telemedicine, medical choices and behaviors must be analyzed taking in consideration not only socio-anagrafic characteristics but also access, skills, and use of ICT.

We talk about perception because the idea we have of Telemedicine is a mix between observation and the mental image that built our awareness. We know that the results of Telemedicine can depend on the relationship that the specific user has with the proposed information system (Dünnebeil et al., 2012), and we want to analyze which factors affect this “image”. If, as Davis (1989) argues, the actual use of a system is influenced by perceived ease and usefulness (cited in Sezgin and Yildirim, 2014), questioning these aspects becomes crucial to support the increase of Telemedicine in Calabria. Furthermore, if success in the use of health services depends on the adoption and acceptance of the service by users (Walter and Lopez, 2008; Holden and Karsh, 2010) it is fundamental to identify these impeding causes.

The scientific purpose of this study is to identify digital, socioeconomic, and cultural inequalities in the access to Telemedicine among University community members during COVID-19, analyzing how digital, socioeconomic, and cultural characteristics affect the perception of Telemedicine, with the final aim to promote Telemedicine in Calabria in post-COVID-19.

To fulfill this study's aims, one main question and five subquestions have guided this study.

Main question: “To what extent do socio-anagraphic characteristics of the University Community members influence their access, skills, and use of ICT; and to what extent does that influence their choices, behaviors, and perception of Telemedicine?”

Sub-questions:

1. To what extent do socio-anagraphic characteristics of the University Community members influence their medical choices and behaviors during COVID-19?
2. To what extent do socio-anagraphic characteristics of the University Community members influence their access, skills, and use of ICT?
3. To what extent do socio-anagraphic characteristics of the University Community members influence their perception of Telemedicine?
4. To what extent do the access, skills, and use of ICT influence the University Community members' medical choices and behaviors during COVID-19?
5. To what extent do the access, skills, and use of ICT influence the University Community members' perception of Telemedicine?

Considering previous theoretical assumptions we define socio-anagraphic characteristics as gender, age, spatial collocation, educational and professional levels, income, family composition, health condition, political and cultural participation, and social life; medical choices and behaviors during COVID-19, as timeliness and how to recognize COVID-19, communication, and treatment of any symptoms (in this case, we are not talking about the use of Telemedicine itself but the medical choices and the medical behaviors that every subject made also offline); perception of Telemedicine as the optimistic or pessimistic impression detected on a pre-tested Likert Scale.

Following, we will describe the project and its aims. We will then describe the material and methods used, focusing on our digital social research that provides web and app surveys with particular technological solutions. Next, we will define the research design, the population, sampling, data collection, and types of data analysis. Further, the main results will be presented and discussed with relevant literature. Finally, we will propose a direction to develop Telemedicine in Calabria and provide an indication for future research.

### 1.3. The project

The project, “Medical Listening and Intervention Unical Online”, aims to address the pandemic emergency by offering an immediate medical listening and consultation channel, as well as surveying and analyzing data related to vaccine prophylaxis and linking them to social variables, such as users' characteristics, choices, and behaviors in the context of health care and Telemedicine. The project stems from the integration of the Life Science and Social Science and Humanities areas and is funded by the Special Supplementary Fund for Research (Italian Ministry University and Research).

The project is developed thanks to the University Health Center of the University of Calabria, in close collaboration with the University Center of Information and Communication Technology and the Departments of (1) Pharmacy, Health, and Nutrition Sciences, (2) Computer, Modeling, Electronic, and Systems Engineering, (3) Political and Social Sciences.

This pilot project is the starting point for a larger project, aiming to develop the service over the entire region. From a long-term perspective, the system may accommodate general biomedical diagnostic services, extending and/or changing the scope of applications beyond COVID-19 contagion.

The project is developed through different objectives, and in this paper, we will discuss only the sociological objective, which is to identify socioeconomic, digital, and cultural inequalities that can affect the development of Telemedicine in Calabria.

## 2. Material and methods: using institutional web and mobile app for web survey

At a time of physical distancing, the research project is developed through remote contact, and, only in some cases, actual in-person medical intervention.

With the advent of the pandemic, the University of Calabria had already swiftly equipped itself with a platform available in web-app and mobile app versions for monitoring access to classrooms and facilities and for contact tracing, useful for managing the flow of attendance and containing COVID-19 contagions. Such platform, called *SmartCampus*, is ideal to develop this Telemedicine project because: (a) it was already used massively by all students, professors, and technical-administrative staff, (b) it could be integrated with additional features, (c) it was designed with a user-friendly interface.

Moreover, a significant advantage was that the platform was available on mobile. In recent years the use of smartphones (even for the simplest everyday tasks) has grown exponentially. From a Telemedicine perspective, there was the rise of so-called m-health (Istepanian et al., 2010; Hampton, 2012), a subsector dedicated to the exclusive use of mobile platforms to offer medical services. Hand in hand with the growth in the use of smartphones (Pettey and van der Meulen, 2012), the use of Telemedicine applications through mobile apps growing (Tachakra et al., 2003). Although some studies highlight that the use of mobile technologies carries the risk of reducing the quality of health care service (Visvanathan et al., 2011), it is also true that the results of a health care service delivered through mobile technologies depend a great deal on the relationship that the specific user has with the proposed information system (Dünnebeil et al., 2012).

In our specific case, where most of the population is made up of students, the use of a mobile application can certainly be considered a relevant methodological choice, overall considering the possibility to use it as a web application. In the following part, we will briefly cover the different stages of designing web and app survey, starting with the technological solution proposed by the IT team.

### 2.1. A technological solution for data collection

The project develops in two main phases: (1) design of software and questionnaires, and (2) data collection and analysis.

The first phase (4 months) was the design, development, and implementation of a software module called “Compass”, as well as the design and pretest questionnaires for two different surveys.

The design of the module included the following:

- The definition of the data representation model to support the storage and subsequent analysis of the data collected by questionnaires.
- The definition of the flowcharts of the questionnaires.
- The design of the specific component for the teleconsultation service.
- The definition of the interfaces of the software components to be integrated under the architecture of the existing application (*SmartCampus*).

The subsequent development phase led to the prototype implementation of the designed software components, their validation, and integration into the *SmartCampus* APP. The software module “Compass” was integrated into the *SmartCampus* APP of the UNICAL, available in a web app and mobile app version for Android and iOS operating systems to the whole University community (students and staff).

The team members tested the prototype version of “Compass” and identified potential improvements to increase usability and ease of use. The feedback received at this stage was implemented, obtaining the final sign-off. The module was then made publicly available within a new version of the *SmartCampus* app released in the app stores.

It is composed of a set of questions that directs the patient to the most useful medical solution (Q1—the effective Telemedicine service), an epidemiological questionnaire (Q2), and a social research questionnaire (Q3). In this article, we will focus on Q3.

Q3 was structured to collect sociological data. On the web app, all the responses to the questionnaire were recorded and reports in a multidimensional format were generated. This allowed us to follow the evolution of data collection in real time.

### 2.2. Web survey design

**Figure 1:**
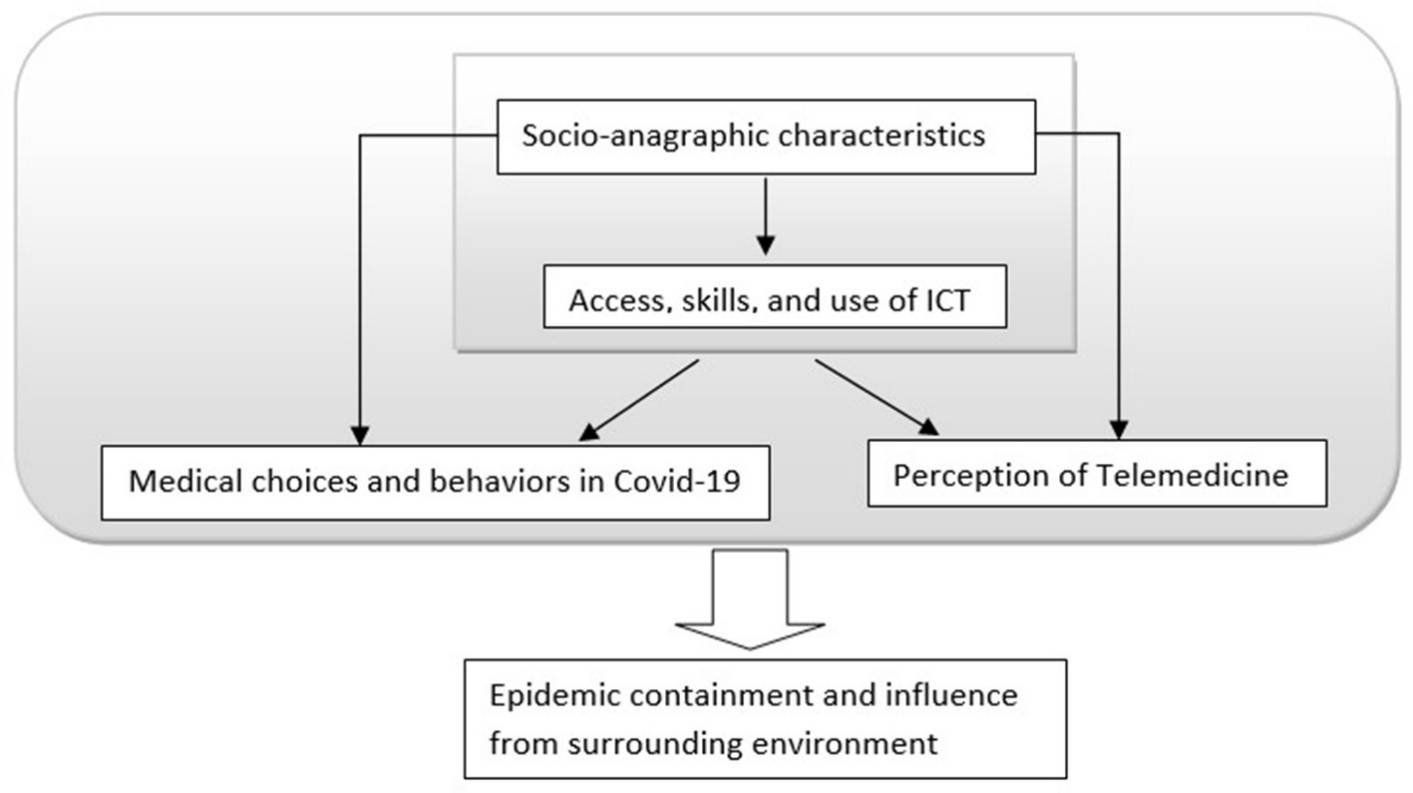
Model of investigation.

The model of investigation represented in Figure 1 will drive the research.

In terms of content, the survey model involved the intersection of the different areas present in Figure 1, assuming a direct influence of socio-anagraphic characteristics and the digital skills of the subjects on the individual choices and behaviors, and the perception of Telemedicine (positive vs. negative). Finally, we explore how the set of these characteristics might affect the surrounding environment. However, this last part of the project is not presented in this paper.

Considering the research set forth and reflecting on group investigation, we have integrated different elements to identify inequalities in the Calabria University context.

We have taken into consideration variables traditionally related to health inequalities (gender, age, spatial collocation, educational and professional levels, income, family composition, health condition) and variables that emerge as relevant in recent research (political and cultural participation, social life) or the specific approach to Telemedicine (access, skills and use of ICT), but also health choices and behaviors during COVID-19 (times and ways to take care of one's health) that can influence the adoption of Telemedicine (Chandra et al., 2011; Oster, 2018; Khairat et al., 2019).

We depart from these main hypotheses:

H1. The socio-anagraphic characteristics affect individuals' medical choices and behaviors during COVID-19. In particular: H1.1 Older people are more concerned^1^ than younger individuals and are better informed; H1.2 Individuals with higher educational and professional levels are better informed than people with lower educational and professional levels; H1.3 Those who live farther from a primary place of care are more concerned than people leaving close to a primary place of care; H1.4 t Individuals with worse health conditions are more concerned and are better informed than individuals with overall better health.
H2. The individuals' socio-anagraphic characteristics affect access, skills, and use of ICT. In particular: H2.1 Younger people have more access, better skills, and make more use of ICT; H2.2 The higher educational and professional levels, the best is the access, skills, and use of ICT; H2.3 High-income earners have more access, better skills, and make more use of ICT than people with lower income; H2.4 Individuals with an active political and cultural participation, or a satisfactory social life, have more access, better skills, and make more use of ICT.
H3. The individuals' socio-anagraphic characteristics affect their perception of Telemedicine. In particular: H3.1 As age increases, so does the optimistic perception of Telemedicine. H3.2 As educational and professional levels increase, so does a favorable perception of Telemedicine. H3.3 As income boosts so grows the optimistic perception of Telemedicine. H3.4 Families with “fragile members” will have a more optimistic perception of Telemedicine than families without “fragile members”; H3.5, Individuals with poor health conditions, will have a more optimistic perception of Telemedicine; H3.6 Individuals with active political and cultural participation or a satisfactory social life, will have a higher favorable perception of Telemedicine than people not meeting these criteria.

H4. Access, skills, and use of ICT affect individuals' medical choices and behavior during COVID-19.

H5. Access, skills, and use of ICT affect the perception of Telemedicine. In particular: H5.1 The higher the access to ICT, the more positive the perception of Telemedicine is; H5.2 Digital skills increase positive perception of Telemedicine.

H6. As increase the use and knowledge of Telemedicine, optimistic perception increases.

This study is partly explicative and partly exploratory. It is explicative as we have derived some concepts from previous literature. Nevertheless, the specificity of this study context (University of Calabria) also required an exploratory lens. In this framework, considering the research questions and the feasibility of the study in terms of time and resources, a questionnaire has been chosen as the primary research method (Brian, 2008).

The design of the social research questionnaire (Q3) takes into account the advantages and disadvantages imposed by the web and app administration (Bethlehem and Biffignandi, 2012). It is highly recommended to use short questionnaires with clear and simple questions and answers. Also, using an app imposes special attention on graphics and display problems that may arise from a mobile compilation (Veltri, 2021). Further, given the project's broader scope and the relatively limited space for sociological research, researchers had to prioritize the most relevant sociological variables, as highlighted in the Telemedicine literature.

The following data have been collected:

- Socio-anagraphic characteristics: age, gender, role in the institution (UNICAL), educational qualification, level of income (self-assessment), area of residence (urban-semi-urban-rural; proximity/reachability place of care), household composition (presence/absence of elderly, children, disabled, individuals with acute, chronic diseases, post-acute situations), personal health conditions (self-assessment), social life (family and friendship relationships, political-social engagement).
- Medical choices and behaviors during COVID-19: timeliness and how to recognize COVID-19, communication, and treatment of any symptoms.
- ICT: access, skills, and use in general and social-health settings (self-assessment).
- Telemedicine: knowledge and evaluation (perception).

In particular, the architecture of Compass allows not to directly ask for gender and age, automatically detected at the moment of access. The questions are attached (Questionnaire_design).

The questions concerning other members of the family are detected in a proxy. This allowed us to explore the digital skills of the family members, furthest from digital environments.

Specifically, in the case of ICT skills, self-assessment of personal and household members' skills was surveyed through a self-anchoring scale with scores from 1=no skills to 10=extremely good skills. This is a simplification necessary for the type of study and the limitation in the number of questions that allow us to detect a piece of information, usually more complex, in a single question.

In the case of Telemedicine, given the complexity and lack of knowledge of the topic, it was provided with a battery with a series of dichotomous responses regarding the services used before analyzing perception. 13 services were considered, and only eight were related to Telemedicine. Not specifically Telemedicine: booking appointments, prescriptions charges, services availability search, comparison of services quality, and prescriptions renewal. Specifically, Telemedicine: entering data for self-assessment of my health, receiving results of tests performed, seeking advice from social-health personnel, receiving post-therapeutic care, providing medical data to health care personnel, receiving information about medical treatments to follow, performing clinical monitoring, and checking the outcome of therapy with the competent physician.

To understand the perception of Telemedicine it was given a selected 4-point anchored Likert scaling technique. These constructed items were useful to stimulate reasoned responses on the topic and provide anchors for memory and knowledge. The points range from 1 = completely disagree to 4 = completely agree, adding an off-scale point dedicated to those who did not know the proposed item. Those who had little or no knowledge about the topic were then excluded from part of the analysis. Referring to the literature and previous research on the topic, we limited the items to 12, all related to the potential advantages and disadvantages that this type of service can offer. To obtain the Telemedicine Perception Index, we calculated the sum of the respondents' scores on each item to correlate it with the other available variables.

The scale, but also the entire questionnaire, was pretested first by social health personnel from different medical areas and then by subjects from the same survey target group. Health personnel helped to construct better items and advised on setting up polarities, students, in particular, helped to clarify some questions, like those on digital skills.

Considering the new instrument (Taherdoost, 2016) and the need to administrate in a limited context (University of Calabria) we assess face (Oluwatayo, 2012) and content validity (Straub et al., 2004), including essential items/questions and eliminating undesirable (Lewis et al., 1995; Boudreau et al., 2001). We follow the following steps:

- An exhaustive literature review to extract the related items.
- The group of social researchers evaluates the appearance of the questionnaire in terms of feasibility, readability, consistency of style and formatting, and clarity of the language used.
- The survey was sent to experts in Telemedicine, social science, and other medical areas.
- Items that are not significant are eliminated.
- Proposal of new items/questions.
- The survey was sent to students, professors, and administrative staff.
- Items that are not understandable are eliminated.
- Proposal of new items/questions.
- For each change repeat the procedure.

#### 2.2.1. Population and sampling

The research was conducted at the University of Calabria (UNICAL) and included students, professors, and technical-administrative staff with access to *SmartCampus* APP. It should be emphasized that the possibility of access to medical services comprises not only those directly involved but also their families.

The population investigated is composed by:

23416 students (registered in the academic year 2021/2022).

854 professors and researchers (on 31/12/2022).

588 directors and technical-administrative staff (on 31/12/2022).

Considering the population and the technological solution adopted, we have opted for self-selection sampling, which is a type of non-probability sampling technique. On the one hand, it is more rapid, and it is adequate for units that are committed to taking part in the study, overall, for using Telemedicine services. There is a risk in terms of self-selection bias (e.g., students who think to have advantages responding desirably) and representativity (e.g., students overrepresented because they have more time or interest).

Self-selection sampling is helpful in this case because it gives the possibility to each *SmartCampus*-users (each member of the studied community) to participate in the investigation and enables them to choose to take part in research on their own accord, volunteer, only because they have a specific interest in Telemedicine or simply in their organization (UNICAL).

To create the self-selection sample, we involve three steps:

- We determine a minimum sample size to pursue representativity.
- We publicize the project and that we need units in different contexts and ways (for Q1, Q2, and Q3).
- We check the relevance of units (only for our questionnaire, Q3).

To determine the sample size, we used this formula:


$$\begin{array}{l} sample\;size=\frac{z^{2\;}\times p\;\left(1-p\right)/e^{2\;}}{1+\left(z^{2\;}\times p\;\left(1-p\right)/e^{2\;}N\right)} \end{array}$$

where:

N = numerosity of the population = 24858

z = Z-score = 1.96 (for 95% confidence level)

e = margin of error = 0.05 (5%)

p = standard deviation = 0.5 (50%)

Determining a sample size of 378 cases.

#### 2.2.2. Data collection

Between February and March 2022, questionnaires were delivered *via* the web or app to the members of the community on an anonymous basis. To promote the initiative, we use:

- Online channels: notices on institutional portals, sharing *via* email, and sharing *via* social networks, both individuals and groups.
- several presentations were held in university classrooms.
- administrative offices were contacted *via* email.
- Directors of the departments were contacted *via* email asking for collaboration.

The university community's attitude toward the initiative was generally positive and proactive. Students made clear their interest in the expansion of UNICAL Telemedicine services, emphasizing that the privacy that comes with remote health services can increase requests for assistance, especially when more sensitive topics are concerned (e.g., gynecological, psychological, chronic problems, etc.).

These meetings and presentations also enabled the researchers to answer any questions the respondents might have in real-time and solve any technical problems that might have arisen (e.g., connection, configuration, updating, etc.). No incentives were used, also if it could be a good idea to do it.

Finally, we collected 489 completed questionnaires related to social research. We check the relevance of units departing from the sample size and role in the institution. The number of units self-selected was enough to continue the analysis, 111 more of the sample size previously calculated. Students constituted the 91% of the respondents, followed by faculty staff (7%), and administrative staff (2%).

#### 2.2.3. Data analysis

The data analysis techniques used involve univariate, bivariate, and multivariate analysis. In particular, we departed from an exploratory analysis to arrive at an inferential analysis. Exploratory Data Analysis (EDA) consented to investigate unexpected relations in a context never analyzed. Inferential analysis consented to verify the hypothesis still exposed. The model of investigation represented in Figure 1 shows how we identified dependent and independent variables and helped to understand the development of our statistical analysis.

Practically we followed three steps:

- Univariate analysis: to derive the data, define and summarize it, and analyze the pattern present in it. We explored each variable separately, distinguishing categorical and numerical ones. Here we reported only non-graphical data for the first frequency distribution in percentages and the numerical mean and standard deviation.
- Bivariate analysis: to find the cause and the relationship between the two variables. In this case, we used graphical data to represent the main results. Scatter plot for relationship numerical-numerical variables, but also non-graphical data like Linear Correlation (r) to represent the strength of a linear relationship between two numerical variables. For association categorical-categorical variables, we selected only the one in which chi-square test results are significant and reported both probabilities of dependency between two categorical variables and bar charts that represent frequency distributions.
- Multivariate analysis: we did not report it because we found no significant results.

## 3. Results: detecting socioeconomic, digital, and cultural inequalities

### 3.1. Socioeconomic conditions

Considering the total number of respondents, 60.3% state that they belong to middle-income households. The remainder declares mainly (33.3%) a low income, while only 6.4% of the sample belong to affluent households. In 37.2 percent of cases, respondents also state that they have experienced economic difficulties in the past 12 months.

The respondents mainly reside in urban (43.4%) or semi-urban (43.8%) areas and still show difficulty in reaching both their primary care medical doctor's office and the nearest emergency room, which can be reached exclusively by private transportation in 69.1 and 62% of cases, respectively.

Even in terms of travel time, reaching the medical facilities that should be of closest proximity does not prove particularly convenient. The questions dedicated to travel time were two: one asks “How long does it take you to get to your primary care physician? (Indicate in terms of hours: minutes) __:___”, the second “How long does it take to arrive at a first aid health center? (Indicate in terms of hours: minutes) __:___”.

On average, the primary care physician is about 20 min away from the respondents' home (with a standard deviation of 35 min), and it takes an average of more than 28 min (with a standard deviation of more than 34 min) to reach a first aid center.

Most households are large (4 people on average) including the elderly (20% of households), children (10.6%), or people with disabilities (9.2%), but above all, 35 percent of the subjects surveyed also include people with acute, chronic diseases or post-acute situations.

### 3.2. Behaviors

Although the sample of respondents mainly includes students, declaring, as expected, excellent or good health (67.5%) and stating to conduct an active social life (56.6%), as many as 47.2 percent of them say they would be concerned immediately if they had symptoms potentially attributable to COVID-19. Obvious symptoms would also trigger initial contact aimed at treatment in 89.6 percent of cases, and only 10.4 percent of subjects would wait for their health condition to worsen.

It is also important to note that 70.3% of respondents would contact their primary care physician and, to a lesser extent, deal with COVID-19 infection by contacting friends or relatives (24.9%), consulting websites (3.9%) or going directly to the emergency room (0.8%).

**CHART 1:**
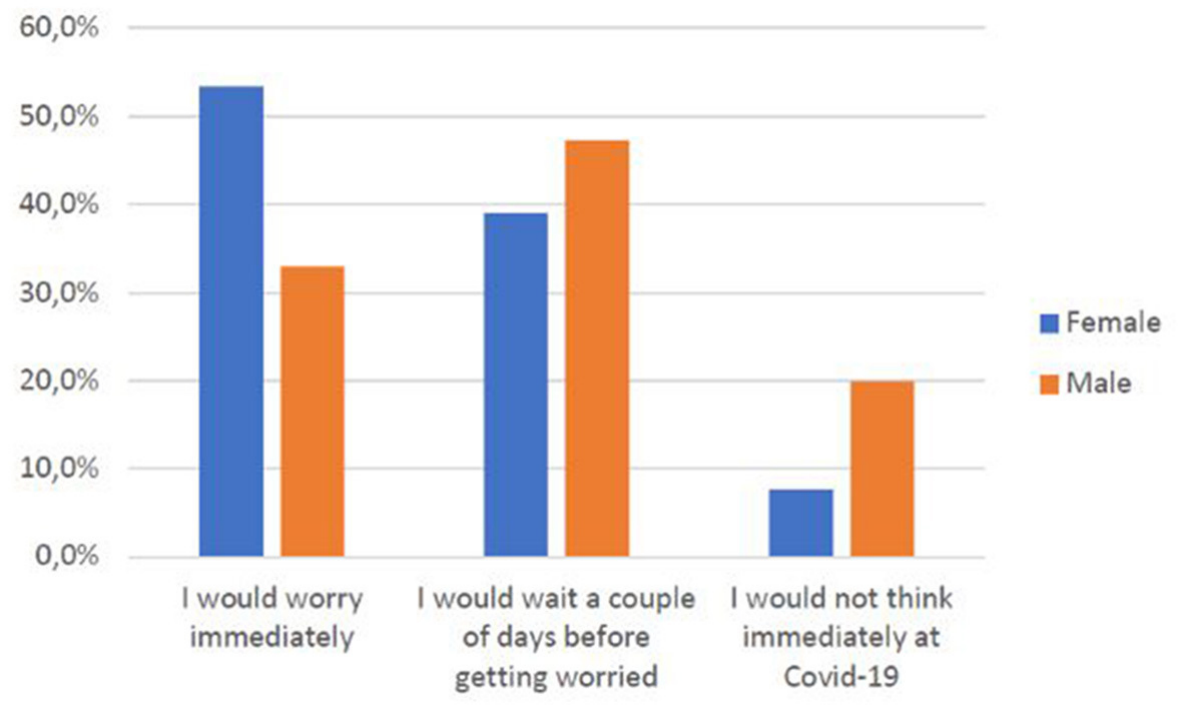
Gender vs. concern related to COVID-19.

**CHART 2:**
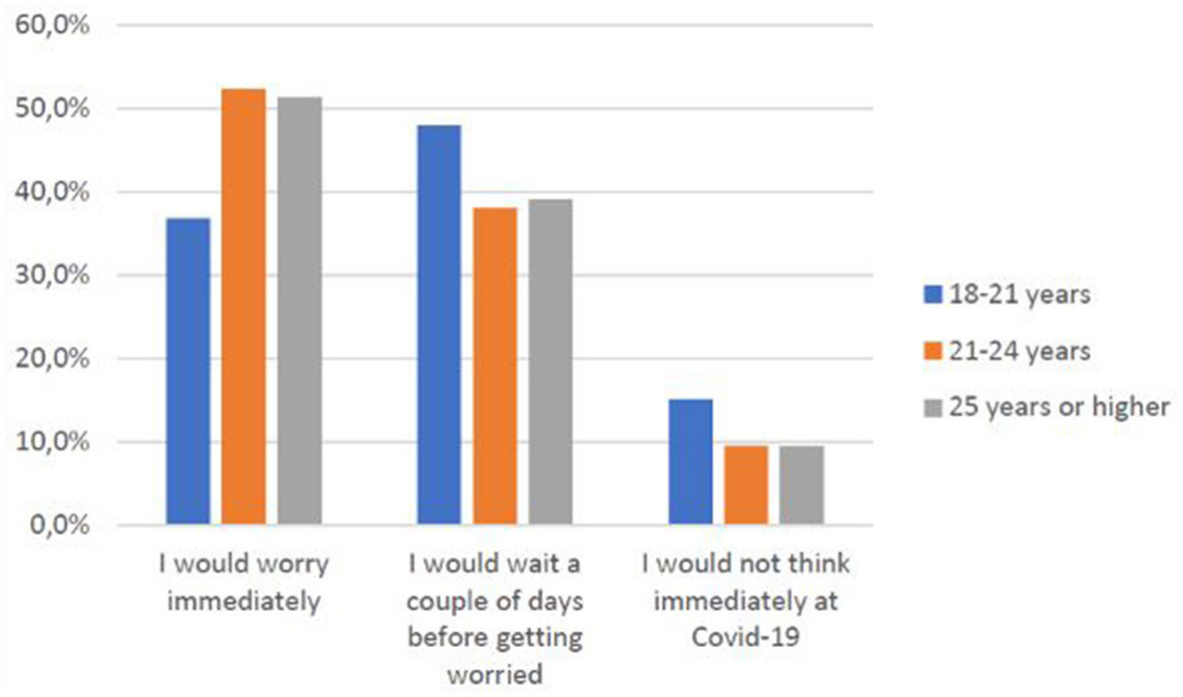
Age group vs. concern related to COVID-19.

Some of the variables emerging from the study were also significant (*p* < 0.05) concerning the subjects' health behaviors. At the onset of the first symptoms, for example, women (Chart 1) and older age groups (Chart 2), in particular, declare to have an immediate concern.

**CHART 3:**
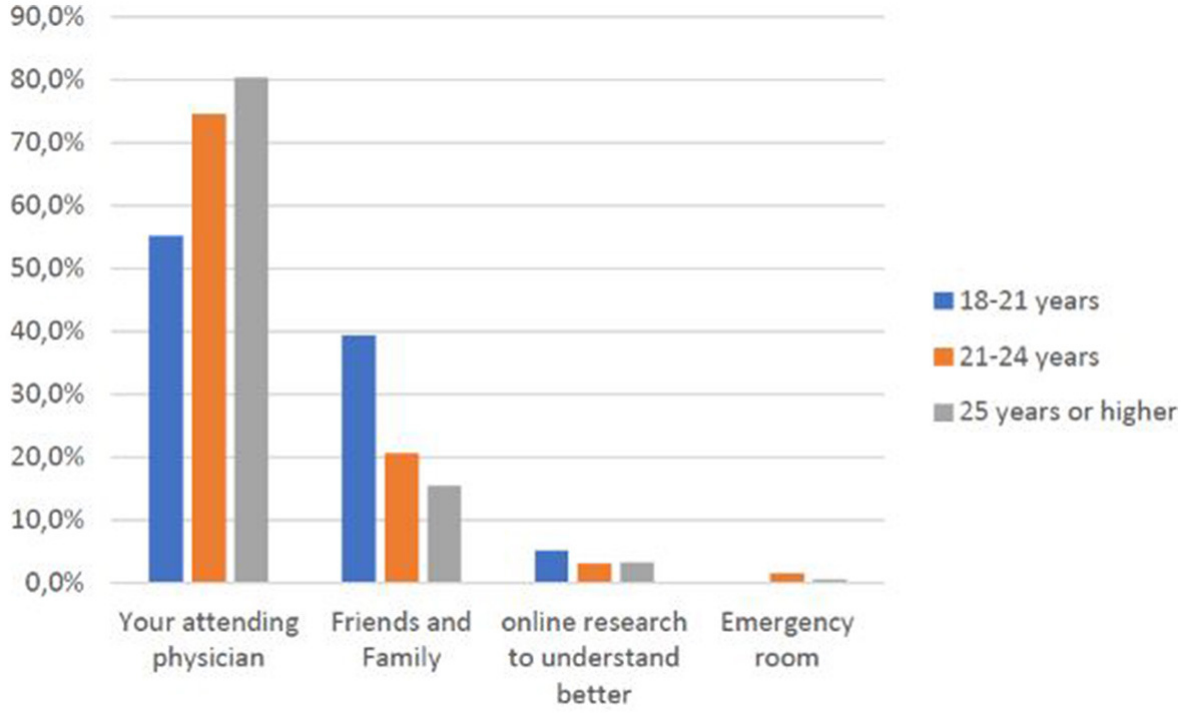
Age groups vs. preferred source of medical help/information.

**CHART 4:**
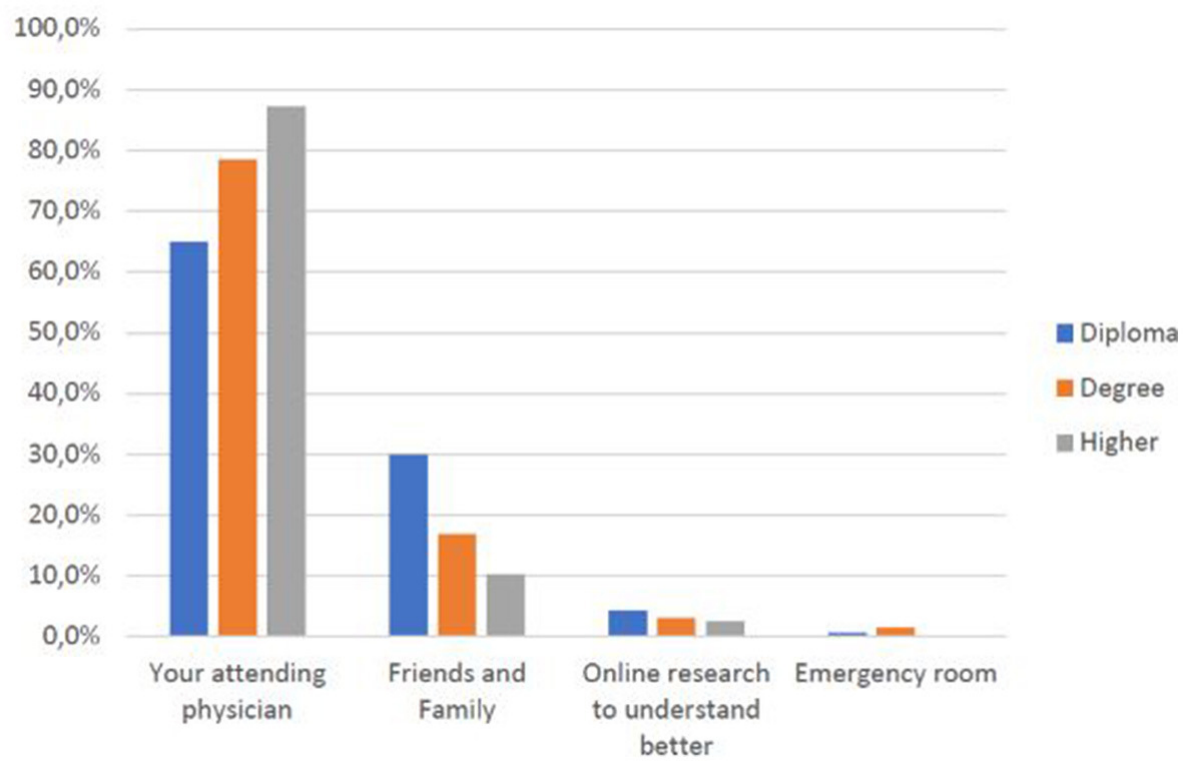
Education level vs. subject preferred source of care.

**CHART 5:**
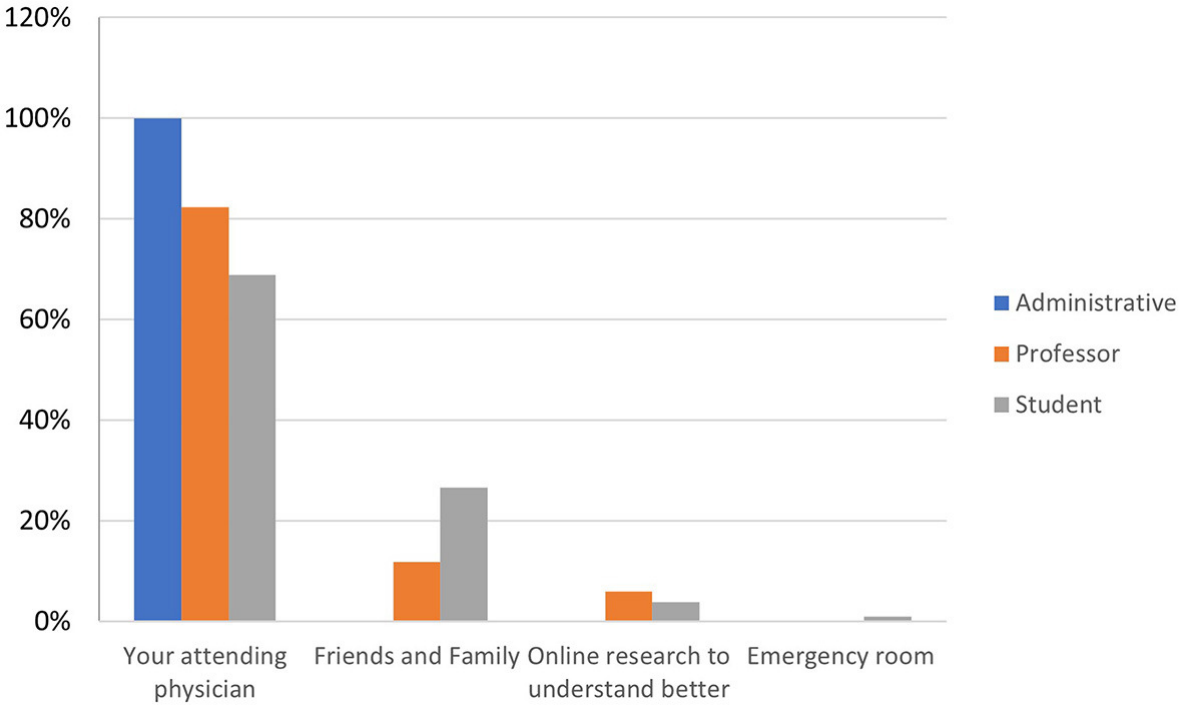
The role played by the subject within the University vs. source of care.

As age (Chart 3) and educational qualifications (Chart 4) increase^2^, so do the shares of those who go directly to their primary care physician rather than consulting websites or relatives and friends, a behavioral difference that is confirmed between students and faculty or technical-administrative staff (Chart 5).

### 3.3. Digital and cultural divide

From a technological and digital point of view, the research shows a particularly active population, which constantly uses digital means of communication in 57.7% of cases, and 40.1% of cases claim to use them often anyway.

The university population has both the right tools (smartphones in 98.8% of cases, PCs or tablets in 98.6% of cases) and a good connection to the Internet (smartphones always connected in 93.9% of cases and PCs or tablets in 61.6% of cases, as well as a stable home connection in 79.3% of cases).

However, a digital skills gap is evident between the respondents and their families^3^: while the university community describes themselves as having good digital skills, assigning themselves an average score of 7.73 out of 10, they state that the digital skills of their family members are much poorer, averaging 5.9.

**CHART 6:**
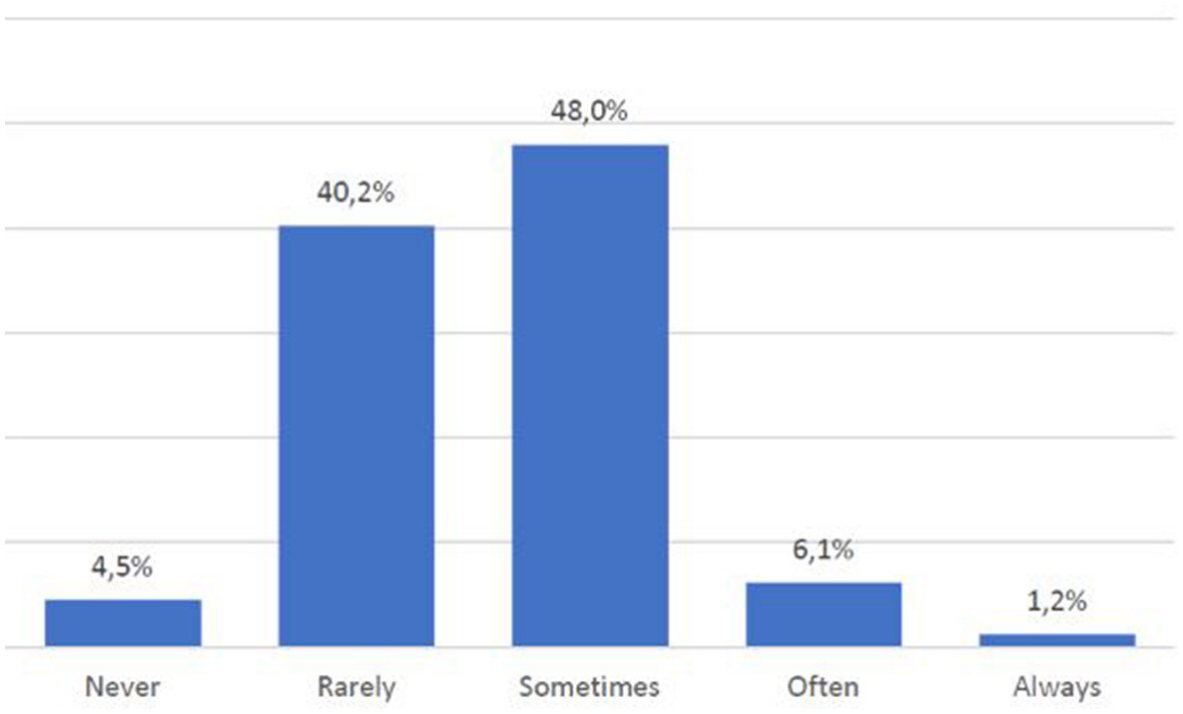
Lack of skills in comprehending online texts.

**CHART 7:**
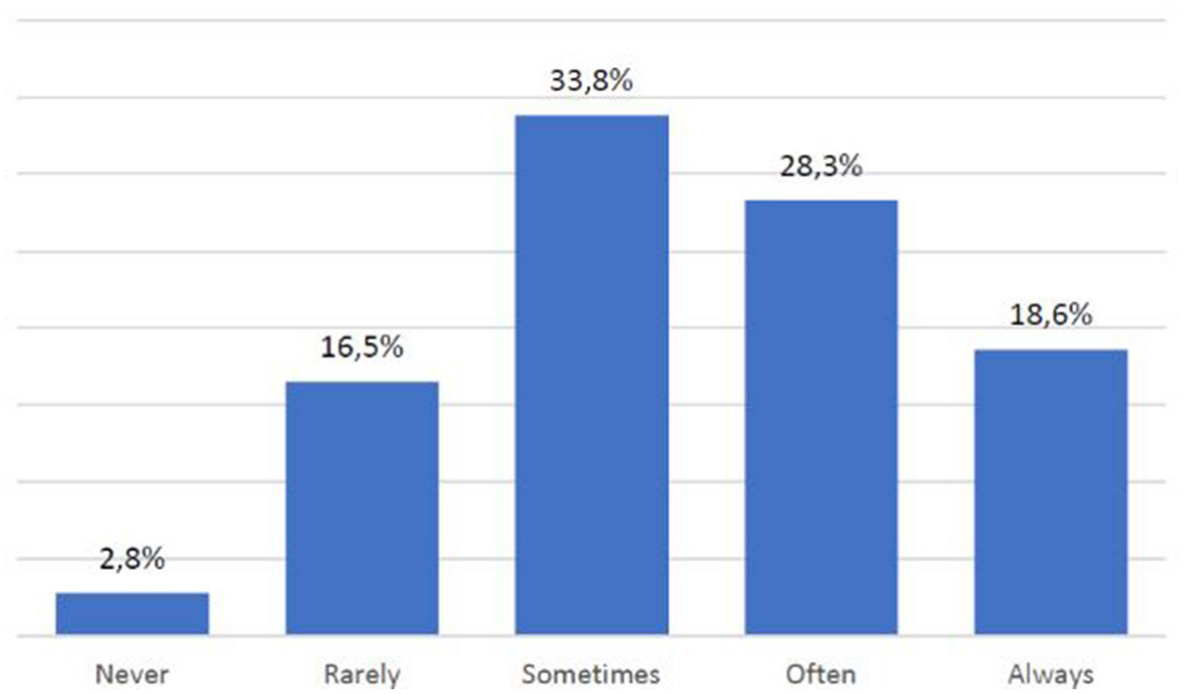
Availability of help to comprehend online texts.

Another particularly significant finding also points out that despite their frequency and ability to use digital media, nearly half of those surveyed (48%) sometimes do not understand the information provided through sites and applications (Chart 6). These are joined by 6% of subjects who often do not understand what is conveyed to them and 1.2% who say they never understand the information provided. In such a context of difficulty, more than half of the respondents (53.1%) also complain that they do not often or always find help to better understand the meanings conveyed by digital media (Chart 7).

### 3.4. Use, knowledge, and perception of Telemedicine

Going into the specifics of Telemedicine, 70.3% of respondents state that they never used it. However, analyzing the subsequent responses shows that, albeit unknowingly, many have used it previously.

Specifically, through ICT, 16.4% received aftercare, 18.4% did clinical monitoring, 34.6% checked the outcome of their treatment, 37.6% entered personal health data for self-assessment of their health, 39.9% received health counseling, 46.6% provided their data to health care personnel, 54.2% received information on treatments to follow, and 79.3% at least received the results of their tests.

As illustrated in Table 1 however, the highest percentages concern online services that cannot be configured as Telemedicine, despite using digital means.

**Table 1:** Percentage of Telemedicine and other online services users.

**Telemedicine services**	**% subjects using Telemedicine**	**Online services not identifiable as Telemedicine**	**% subjects using services not identifiable as Telemedicine**
Entering data for self-assessment of my health	37.6	Booking appointments	75.5
Receiving results of tests performed	79.3	Prescription charges	68.3
Seeking advice from social health personnel	39.9	Services availability search	90.8
Receiving post-therapeutic care	16.4	Comparison of services quality	63.6
Providing medical data to healthcare personnel	46.8	Prescriptions renewal	36.2
Receiving information about medical treatments to follow	54.2		
Performing clinical monitoring	18.4		
Checking the outcome of therapy with the competent physicians	34.6		

Focusing on the analysis of the respondents' perceptions of Telemedicine, a generally positive view emerges, although, precisely because of the lack of knowledge on the subject, there is a high (55.6) percentage of those who fail to express an opinion on the different features that characterize it.

Most respondents agree that Telemedicine is a valuable option to empower citizens (78%) and to reach even those who live in peripheral areas (67.7%), guaranteeing constant clinical monitoring (62%); more than half of the respondents, moreover, say they are not afraid of possible problems regarding the protection of personal data (54.9%).

### 3.5. The award

**CHART 8:**
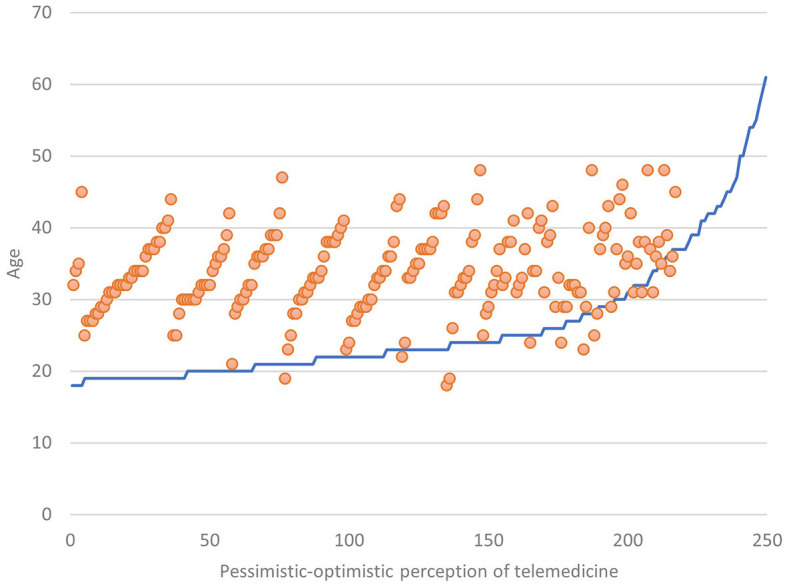
Perception of Telemedicine vs. age.

**CHART 9:**
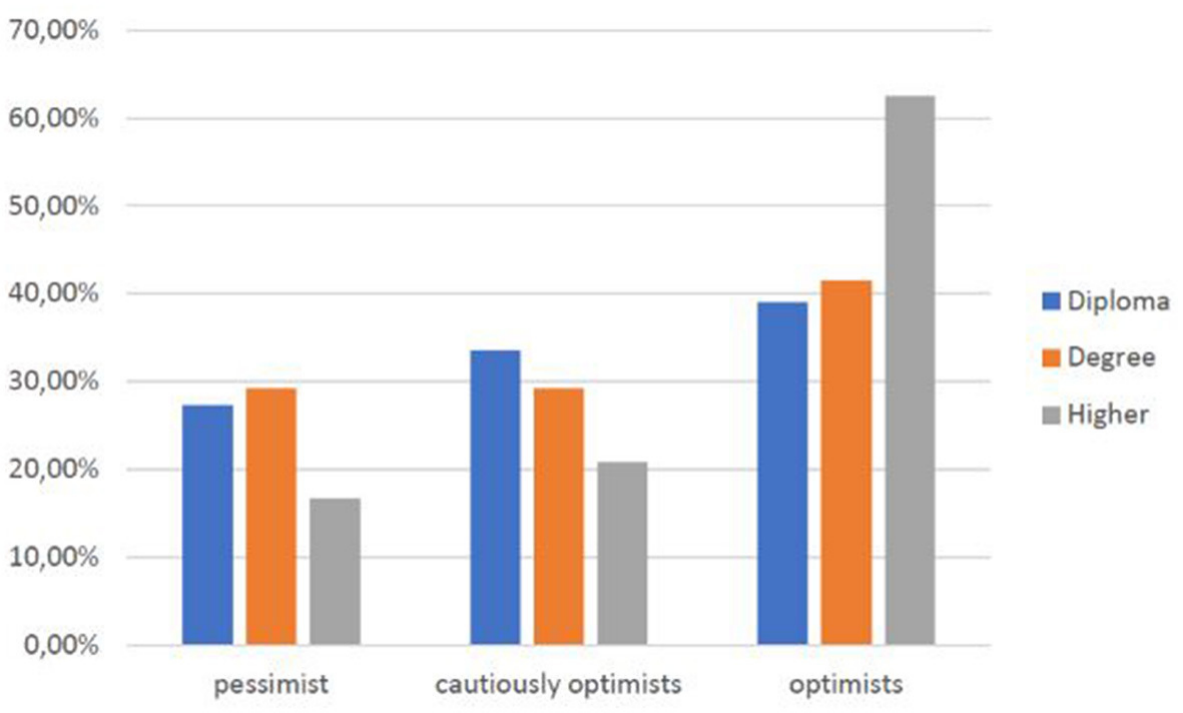
Perception of Telemedicine vs. education level.

Analyzing the sub-sample (44.4%) of those who have a clear opinion about Telemedicine, it becomes evident that as age increases, there is also a slight increase of an optimistic perception toward Telemedicine (*r* = 0.22) (Chart 8), and the same happens when educational qualification increases (*p* < 0.05) (Chart 9).

**CHART 10:**
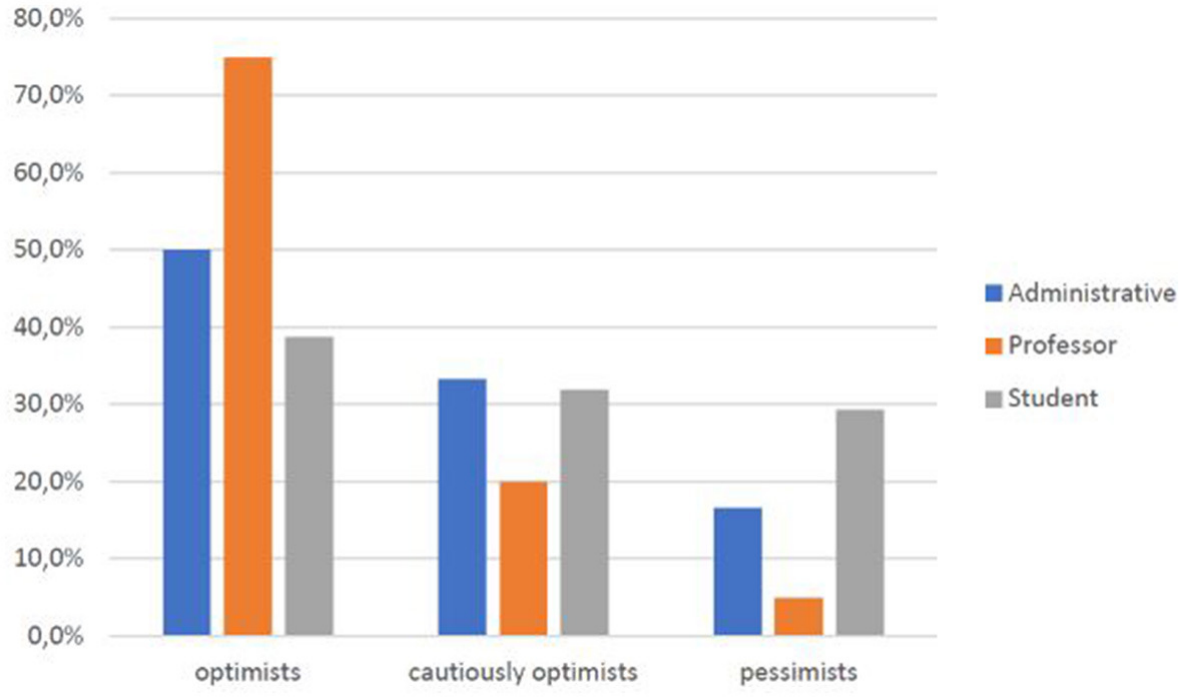
Perception of Telemedicine vs. role at UNICAL.

**CHART 11:**
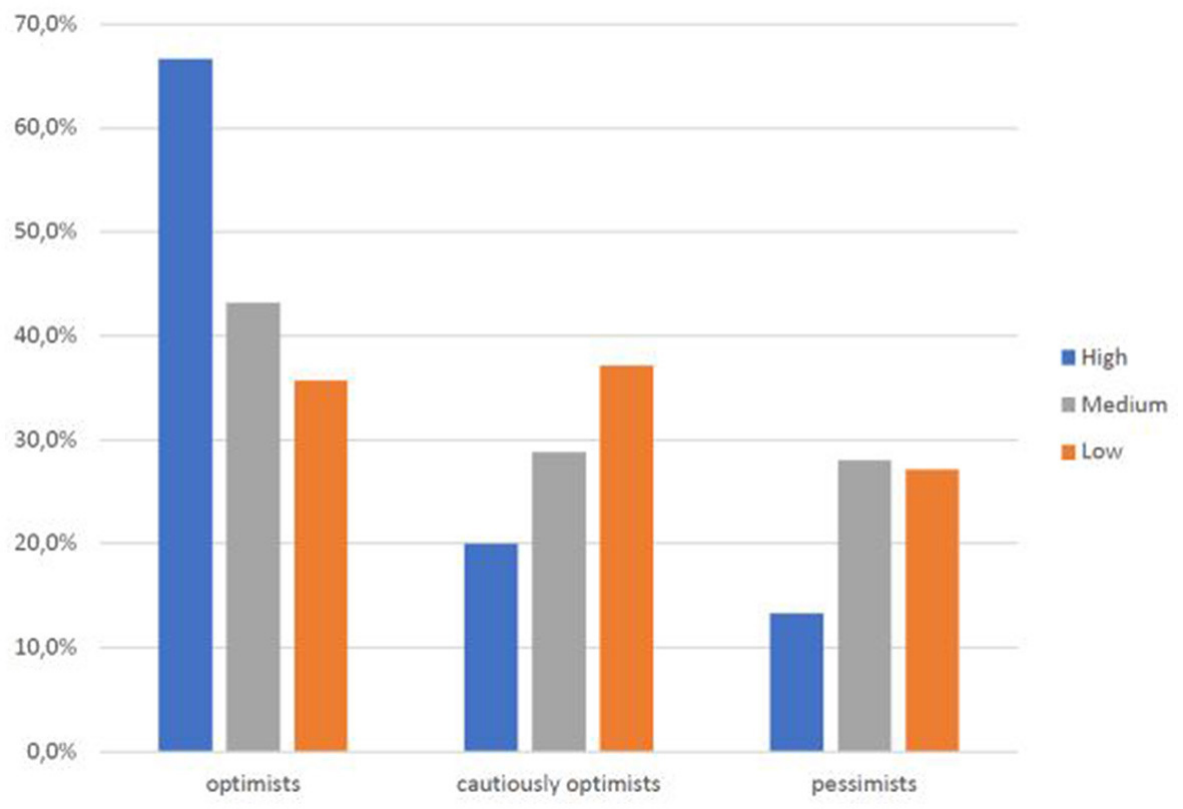
Perception of Telemedicine vs. income level.

Within the university community, the faculty is most optimistic (60% versus 30.9% of students and 16.7% of technical-administrative staff) (Chart 10), a trend that also emerges in those who report a higher income (60% versus 30% of those with low incomes and 31.8% of those who report a medium income) (Chart 11). Thus, the positive view of this type of innovative medical service is also related to socioeconomic level.

A positive perception of Telemedicine also emerges among those whose household includes elderly people or children; on the contrary, it does not emerge from households in which there are people with disabilities or illnesses.

The positive perception of Telemedicine is also found to be significantly associated with the possession of a stable home connection and the daily use of technological and digital means (*p* < 0.05), while there is no evidence of substantial differences in terms of skills possessed by respondents or their families.

**CHART 12:**
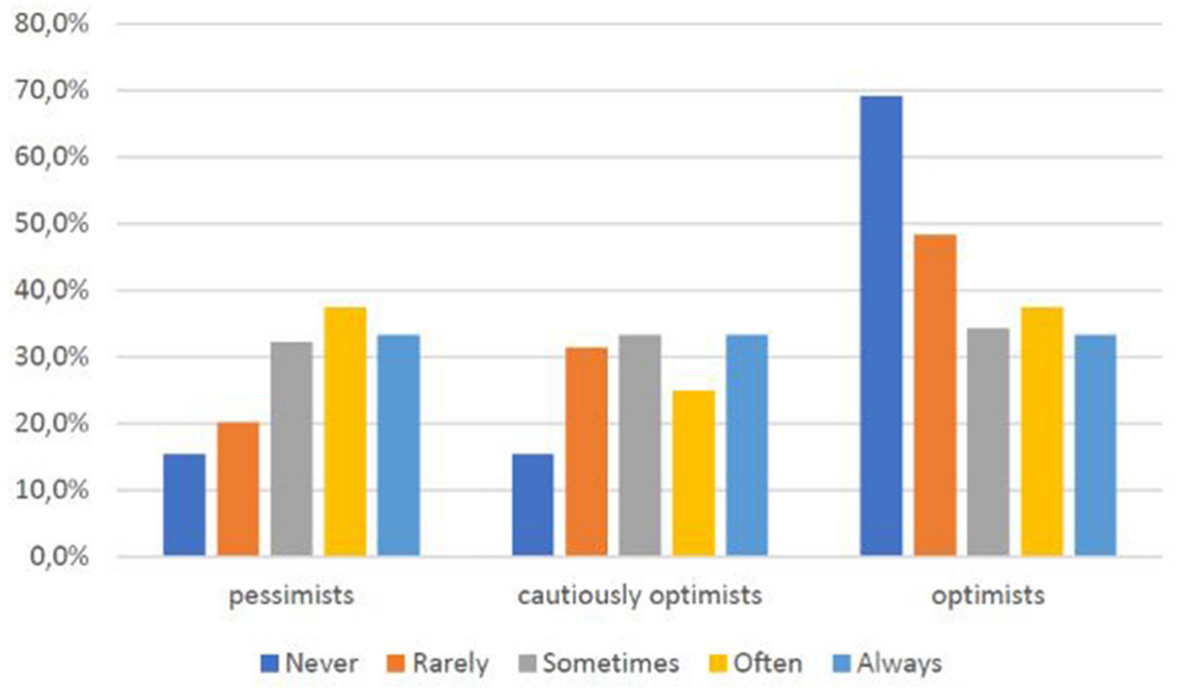
Perception of Telemedicine vs. lack of online text comprehension.

On the other hand, the relationship between difficulty in understanding ICT means and the degree of optimism toward Telemedicine is also found to be significant: those who can understand the tools almost always turn out to have a more positive view of Telemedicine (Chart 12).

**CHART 13:**
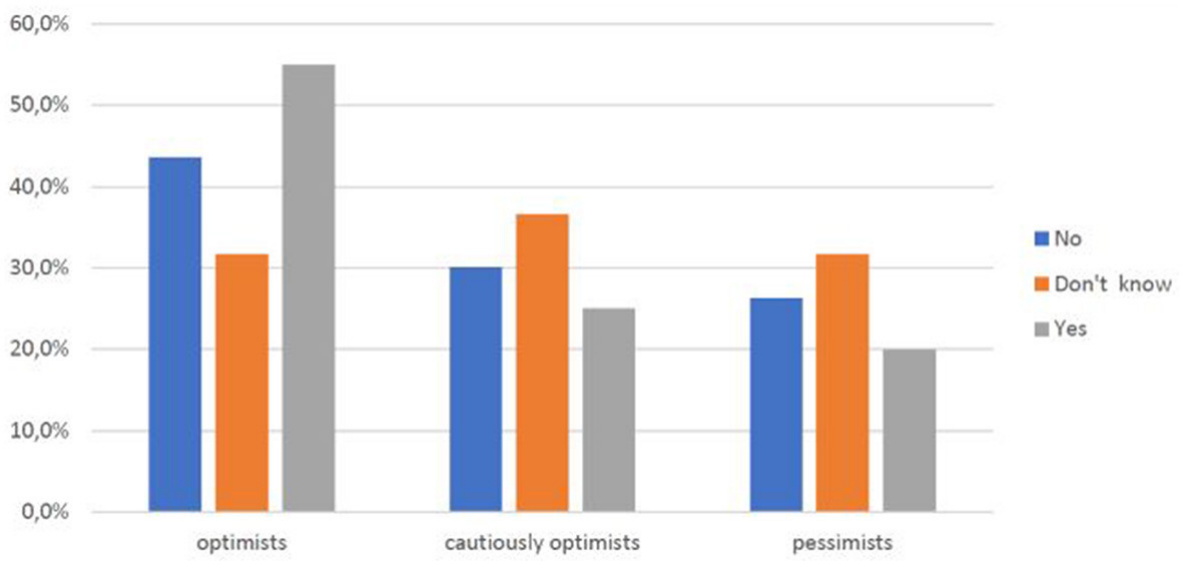
Perception of Telemedicine for Telemedicine use.

Those who say they have already used Telemedicine services are also the most optimistic (Chart 13).

## 4. Conclusion: driving medical innovation process in Calabria after COVID-19

Digital social research identifies digital, socioeconomic, and cultural inequalities to access Telemedicine among the university community during COVID-19. We have analyzed how digital, socioeconomic, and cultural characteristics affect the use, knowledge, and perception of Telemedicine, with the final aim to promote Telemedicine in Calabria in post-COVID-19.

The research results show that digital, socioeconomic, and cultural characteristics of the sample influence perception of Telemedicine, but how?

Below, we present our conclusions based on our collected findings. We do so accordingly to our investigation model (Figure 1) and questions:

(1) *To what extent do socio-anagraphic characteristics of the University Community members influence their medical choices and behaviors during COVID-19?*

Hypothesis 1 “The socio-anagraphic characteristics affect individuals' medical choices and behaviors during COVID-19” is verified only in part.

As Chart 1 shows, female is more concerned about COVID-19, but we don't find a significant relationship between the rapidity of asking for help and the mean of information used.

As Chart 2 shows, older people are more concerned related to COVID-19 and good medical behavior (going directly to their primary care physician), but there is no evidence of the rapidity they take care of. This confirms specific hypothesis H1.1.

As Charts 4, 5 show, higher educational and professional levels have good medical behavior (go directly to their primary care physician), but there is no evidence of the rapidity they take care of or of the concern related to COVID-19. This confirms specific hypothesis H1.2.

Finally, we have not found a significant association between residence in urban or not urban areas or with means of transportation and time to arrive at the first medical help center, so H1.3 is not confirmed. There is no evidence of the relationship between medical choices and behaviors with income levels or families' dimensions and health conditions, so hypothesis 1.4 is not confirmed. Also, political, cultural, or social life is not related to medical choices and behaviors.

(2) *To what extent do socio-anagraphic characteristics of the University Community members influence their access, skills, and use of ICT?*

Hypothesis 2 “The individuals' socio-anagraphic characteristics affect access, skills, and use of ICT” is not verified. There are no evident inequalities related to these dimensions.

We cannot find evidence that access, skills, and use of ICT are related to gender, age, spatial collocation, educational and professional levels, income, family composition, health condition, political and cultural participation, and social life, so H2.1, H2.2, H2.3, H2.4 are not confirmed. In particular, the hypothesis that active political and cultural participation or a satisfactory social life influences access, skills, and use of ICT is in contrast with Heponiemi's (2020) research results.

Never less, the exploration of the data consent to detect interesting results.

Also, if the majority of the sample has optimal access (over 98% possession of digital tools, 93.9% internet mobile access,79.3% home connection) and use (57.7% constant use sum to 40.1% often use) of ICT, a digital skill gap emerges between respondents and families: on our scale respondent assess themselves 7.73/10 while family members are evaluated 5.9/10.

Also, the influence of cultural characteristics emerges not from socio-anagraphic variables but from the comprehension of texts: 55.2% of the sample does not understand digital content (sometimes, often, or never). The worst evidence is that, in such a context of difficulty, 53.1% of them find no help (often or always). So, we can say that cultural characteristics (in this case, in terms of competence) influence skills and use of ICT because people admit to not understanding digital content and, subsequently, they cannot use it well.

(3) *To what extent do socio-anagraphic characteristics of the University Community members influence their perception of Telemedicine?*

Hypothesis 3. “The individuals' socio-anagraphic characteristics affect their perception of Telemedicine” is partly verified.

Only 29.7% of the sample declare to have used Telemedicine, also if 79.3% have used at least one Telemedicine service. Unfortunately, because of the lack of knowledge on the subject, there is a high (55.6) percentage of those who fail to express an opinion on the different features that characterize Telemedicine. Still, we can make inferences departing from people who declare to know it.

As age (Chart 8), educational level (Chart 9), professional level (Chart 10), and income (Chart 11) increase, it also increases an optimistic perception toward Telemedicine. So H3.1, H3.2, and H3.3 are confirmed.

H3.4 is partly verified because a positive perception of Telemedicine emerges among households, including older adults or children, but not disabilities or illnesses. H3.5 and H3.6 instead are not confirmed, the last one in contrast with Heponiemi et al. (2020) research results.

(4) *To what extent do the access, skills, and use of ICT influence the University Community members' medical choices and behaviors during COVID-19?*

Hypothesis 4. “Access, skills, and use of ICT affect individuals' medical choices and behavior during COVID-19” is not verified.

There is no evidence of the influence of ICT on medical choices and behavior.

(5) *To what extent do the access, skills, and use of ICT influence the University Community members' perception of Telemedicine?*

Hypothesis 5. “Access, skills, and use of ICT affect the perception of Telemedicine” is partly verified.

A stable connection and frequent use of ICT are associated with a positive perception of Telemedicine, but there is no evidence of skills. H5.1 is verified; contrary H5.2 is not.

As Chart 12 shows, another interesting result that emerged from the exploration of data is that comprehension of digital content is associated with an optimistic perception of Telemedicine.

Hypothesis 6. “As increase the use and knowledge of Telemedicine, optimistic perception increases” is verified. As Chart 13 shows, those who say they have already used Telemedicine services are also the most optimistic, highlighting how knowledge of the tool helps redefine perceptions of it.

The multivariate analysis doesn't underline significant results that can help us to detect latent factors or natural groups that are more or less inclined to use Telemedicine.

In conclusion, we verified that socio-anagraphic characteristics affect individuals' medical choices and behaviors during COVID-19, underlining gender and age influences, but also the influence of education and employment levels. On the contrary, there are no differences in Calabria University Community related to the area of residence, the health conditions of the households, or the social habits of individuals.

The individuals' socio-anagraphic characteristics don't affect access, skills, and use of ICT, also if there is a digital skill gap between respondents and their families. The worst evidence is the lack of comprehension of digital content and the lack of help in understanding it.

Results underline that individuals' socio-anagraphic characteristics affect the perception of Telemedicine: as age, educational level, professional level, and income increase, it increases an optimistic perception toward Telemedicine. Additionally, households with older adults or children have a more positive perception of Telemedicine.

Access, skills, and use of ICT don't affect individuals' medical choices and behavior during COVID-19, but affect the perception of Telemedicine, optimistic if there is a stable connection and a frequent use of ICT. Overall, as increase comprehension of digital content, optimistic perception increases.

Finally, as increase the use and knowledge of Telemedicine, optimistic perception increases, and this is an important finding to develop Telemedicine in Calabria.

## 5. Discussion

Telemedicine can overcome inequalities (Williams, 2003), but it cannot be made if there remain inequalities in access to technology (Cotton and Gupta, 2004; DiMaggio et al., 2004). Therefore, an environment ready to accommodate digital innovation is the essential foundation to foster medical innovation in Calabria after the COVID-19 pandemic.

The pandemic highlighted how new technologies can solve problems that would have otherwise remained unsolved (e.g., Cipolla et al., 2021). The risk incurred in other epidemics that preclude physical contact and the need to reduce the time and distances related to medical (as well as psychological or social) intervention should prompt us to question how to overcome the limitations imposed by the physical environment. Digital social research and digital medicine services are the means for us to do so.

As highlighted by this paper, the “Medical listening and intervention Unical online” project is not just an excellent achievement for the Telemedicine services provided in Calabria. Still, it is outstanding for coupling this offering with digital social research. Furthermore, the interdisciplinary approach—to which IT experts substantially contributed—enabled us to respond to an immediate, multidimensional, and complex need, in which topics related to subjects' behaviors, choices, and experiences did not remain unnoticed.

The results show that the Calabrian university community is composed of middle-income households, but during COVID-19, 37.2% experienced economic difficulties (Gray, 1982; Link and Phelan, 1995). Calabria, for subjects, is a complex context in which most people have difficulty reaching their primary care medical doctor's office and the nearest emergency room, and the travel time to reach them is always too high (Seidel et al., 2006). The lack of healthcare providers in the South of Italy can be a problem (Brems et al., 2006; Wilson et al., 2009). Also, suppose most of the sample live in urban or semi-urban areas in this context. In that case, Telemedicine could offer great advantages economically, logistically, and concerning time management, benefits that address territorial inhomogeneity and the lack of infrastructure or social healthcare providers (Sood et al., 2007) that we can observe in Italy and overall, in South and Calabria in particular (Giarelli and Giovannetti, 2019).

The sample is also composed of large households, including elderly, children, and people with disabilities or acute, chronic diseases or post-acute situations (Wang and Liu, 2006), for whom the services offered by the project would provide additional benefits (Italian Ministry of Health, 2012), also if results underline that they have no awareness of this. This is a significant result.

The sample mainly comprises students with good health and an active social life. The concern related to COVID-19 is 47.2%, but in 89.6% of cases, apparent symptoms trigger initial contact aimed at treatment, and the first person contacted would be their primary care physician. This evidence shows concerned, careful, and responsible young people, alert to the onset of COVID-19 symptoms, contrary to what media and common-sense underline during COVID-19.

As still detected in other research (Barber and Kim, 2021; Datta and Eiland, 2022) females and older have more concerns related to COVID-19. As age, education, and professional level increase, good information addresses personal physicians. Age is an ascribed variable, but we can reflect on the importance to strengthen education (Luo et al., 2021) and a labor market that permits—also in Calabria—to achieve a high professional goal and better medical choices and behavior (Paige et al., 2018).

In general, the results indicate trust in the National Health System and awareness of both the ineffectiveness of self-medication and the need to prevent the overloading of first aid stations. In this context, Telemedicine would reduce the burden on primary care physicians (Battineni et al., 2021) who, during the pandemic, had difficulties dealing with their workload (Gagnon et al., 2006). Telemedicine offers easier, more immediate, and direct contact and avoids physical contact, thus preventing the spread of some diseases (Sood et al., 2007). This tool is not only a proper means of medical consultation and intervention but also reassures the patient, who no longer finds himself in a prolonged state of uncertainty due to prolonged waiting times (Italian Ministry of Health, 2012).

So, in the first part of the analysis, we have demonstrated that “socio-anagraphic characteristics of University Community members influence their choices and behaviors” and we have identified the elements on which we can intervene.

Then we have seen that “socio-anagraphic characteristics do not influence access, skills, and use of ICT”. Maybe because most of the population has yet excellent access to ICT and uses it every day (Lechman, 2015), we cannot identify the association with socioeconomic and cultural characteristics (Tobishima, 2020). It is essential to underline that there is a relevant difference between the university community and their families. This result reflects on the specificity of the sample, who belongs to a favored context (University), and on the necessity to extend the research to different contexts.

On the other hand, contrary to what we can think, the analysis of the ICT dimension shows a population who have relevant problems in text comprehension (Beaunoyer et al., 2017) and also in finding someone helps. This can be a great challenge for the University of Calabria: it is not sufficient to have access, use, and have digital skills if we cannot comprehend digital content, and this is a problem for all educational institutions (HEIs) in the territorial area of Calabria. Action is needed both to increase basic digital skills in the general population (European Commission, 2022) and to strengthen autonomy in understanding written material and how informatic systems also work (Bagozzi et al., 1992).

Finally, “socio-anagraphic characteristics of the University Community members influence their perception of Telemedicine”, and this is why it is necessary to overcome inequalities. We have already seen that the use of Telemedicine depends on the usability of the information system (Dünnebeil et al., 2012), which is strictly connected to the perception we have of it (Altmann et al., 2022).

Results show that households that include elderly people or children have an optimistic perception of Telemedicine, but the same doesn't happen for households in which there are people with disabilities or illnesses. It is really important to inform people about the advantages offered by Telemedicine, and overall, households can receive more benefits from it (Annaswamy et al., 2020).

The research shows an increase in optimistic perception of Telemedicine, increasing age, educational level, professional level, and income (Luo et al., 2021), confirming the importance of investing in educational and labor policies that permit facing inequalities. On the contrary, the research doesn't confirm that an active social life influences the perception of the benefits of ICT (Heponiemi et al., 2020). Maybe because a context such as Calabria doesn't offer a “social life” comparable to the Finnish one.

It is not confirmed that “access, skills, and use of ICT influence the University Community members' choices and behaviors”, but “access and use of ICT are associated with an optimistic perception of Telemedicine”. Maybe we don't detect an association with skills because we have not deepened the different digital skills of the population (Pinciroli et al., 2011), but the main result is that comprehension of digital content is associated with an optimistic perception of Telemedicine. So, it is necessary to invest in digital education.

Finally, we find that using Telemedicine help to have a more optimistic perception, so people who use it and are aware of doing it, understand the great advantages it takes. If success in the use of health services depends on the adoption and acceptance of the service by users (Walter and Lopez, 2008; Holden and Karsh, 2010) it is indeed necessary to act on awareness of addiction to socioeconomic and cultural aspects.

The unawareness is the most important result detected. The unconscious involvement of users in Telemedicine can be a first lever to increase its use. It is important to raise awareness of its opportunities and advantages (Yellowlees, 2005). It is an issue that falls squarely within cultural inequalities, which do not even allow the recognition of Telemedicine as a tool used in daily life. Unawareness is the first challenge to solve, as—to take advantage of an opportunity—it is necessary to know its merits and drawbacks.

Exploring the last two areas (ICT and Telemedicine) has allowed us to have a greater awareness of a service that is still little known to users, especially in a region where the development of digital medicine is in progress. As Andreassen and Dyb (2010) underline ≪relation between IT and social inequalities in health is constraining as well as insufficient to explain the persistence of health inequalities in digitalized western societies≫.

This study suggests how limited penetration of technological advances in the population must be addressed primarily by overcoming sociocultural and economic barriers and developing knowledge and understanding of digital environments. This can be an important way to lay the foundations for an achievable process of digital medical innovation in Calabria.

The proposed technological solution (“Compass”) would make Telemedicine a useful tool for the National Health Service, by improving its efficiency, reducing time and costs, and increasing flexibility. The long-term deployment of the app would also improve communication between physicians and patients and between different healthcare providers, facilitating specialist consultations and prescriptions. The versatility of the project is made evident by its long-term prospects and potential: in the future, the system developed for the current situation can easily be repurposed for general diagnostics, independently of COVID-19, and extend and/or modify the medical services field by transforming, in Calabria, the dialogue and exchange of information amongst physicians, specialists, pharmacies and patients.

In conclusion, we want to underline that, departing from these results, if National Health Service (NHS) and the regional government want to promote an achievable process of digital medical innovation in Calabria, it is crucial to overcome inequalities through adequate public policies and to strengthen partnerships with higher education institutions (HEIs), to overcome socioeconomic, cultural, and digital gaps in Calabrian population.

Digital social research proved to be an indispensable tool, both for carrying out research in the COVID-19 period, and for obtaining necessary findings to develop Telemedicine in Calabria, identifying factors that may affect the service. The research showed that the progression of digital medicine in Calabria and the development of the Calabrian health network should not be driven by technological or infrastructural development but by socioeconomic and cultural development to reach a better understanding and use of digital resources. If the fruition of a digital service is closely linked to the user's relationship with the proposed information system (Dünnebeil et al., 2012), the ability and willingness of users to make use of an innovative service affect the actual realization of the possible medical-health innovation.

This pilot research presents certainly some limitations: it is a proof of content, and the questionnaire and the scale must be better tested and developed. In particular, we must keep all dimensions we have detected with few questions. It has been necessary because of the time and requests of the interdisciplinary group of research, considering the different aims that have the entire pilot project. It will be important to deepen concepts and to check new instruments in the territorial area of Calabria. It could be important also to develop multivariate analysis. Also, the population investigated must be expanded, both to limit sample bias and to foster the medical innovation process in the entire region.

Future research can take into consideration also attitudes of physicians, specialists, scholars, and facilities in the area, who are also closely involved in the use of digital platforms. It could be useful to deepen the research with other methods, both quantitative and qualitative. We are thinking about developing UX research (Barnum, 2019; Zheng et al., 2022) and cyberethnography (Ward, 1999), giving a more profound interpretation of the present results and suggesting new directions for research.

## Data availability statement

The datasets presented in this article are not readily available because restrictions apply to the original data under the GDPR regulation. Requests to access the datasets should be directed to ltaddei@unisa.it.

## Ethics statement

Ethical review and approval was not required for the study on human participants in accordance with the local legislation and institutional requirements. The patients/participants provided their written informed consent to participate in this study.

## Author contributions

LT is the first author and the corresponding author. She has been responsible for the organization of the writing and the design of the concept of the article. She has designed the social research questionnaire and she is responsible of the analysis and interpretation of the results. FM is the medical director with experience in conducting clinical-epidemiological studies, he dealt with the analysis of data hesitated by the Telemedicine service for suspected contagion from COVID-19 and for the side effects of the vaccine prophylaxis activity. EP is scientific manager of the social research, he constantly monitored the progress of the survey during data collection, planning, organizing and participating in periodic meetings with the team members involved. TG has followed the progress of the investigation by participating in periodic meetings with the research team. EP and TG supported LT in the design of the social research questionnaire and in the analysis and the interpretation of the results, contributing to the writing of the article. AM and RM helped conceive the project and provide technical and organizational support to write the article. All authors contributed to the article and approved the submitted version.

## Footnotes

^1^It means that they worry before if they have symptoms that could be traced to COVID-19 (question 16) and that they will contact someone immediately (question 18).

^2^Educational levels were 4 but became 3 because in “1) elementary/middle school diploma” there are no cases. It remains a high school diploma, bachelor's or master's degree, or postgraduate (e.g., doctorate, specialization).

^3^The digital skills of families have been surveyed by a proxy question, necessary for the cost/benefit tradeoffs despite the conscious risk incurred (Cobb, 2018).
