# Supplementary material for: Contributions of digital social research to develop Telemedicine in Calabria (Southern Italy): identification of inequalities in post-COVID-19
Source: Front Sociol. 2023 May 9;8:1141750. doi: 10.3389/fsoc.2023.1141750 (PMC10204871; doi:10.3389/fsoc.2023.1141750)
Supplement: Supplementary file 1 [file Data_Sheet_1.pdf]

Questionnaire design – Q3
"Medical Listening and Intervention Unical Online"

Role in the institution:
1) student
2) administrator
3) lecturer

Educational Qualification:
1) elementary/middle school diploma
2) high school diploma
3) bachelor's or master's degree
4) postgraduate

How would you define your household income:
1) high
2) medium
3) low

Do you have economic difficulties in the past 12 months?
1) yes
2) no

You are resident in an area:
1) urban
2) semi-urban
3) rural

How long does it take you to get to your primary care physician?
(Indicate in terms of hours: minutes) __:___

Can you reach your attending physician's office?
1) by public transportation
2) by public transportation, but only at certain (very reduced) times
3) only by private transportation

How long does it take you to arrive at the primary care health center?
(Indicate in terms of hours: minutes) __:___

Can you reach a first aid health center?
1) by public transportation
2) by public transportation, but only at certain (very reduced) times
3) by private vehicle only

How many people do you live with?
n __

They are present:

	Yes	No
elderly (over 65 years old)		
children (under 12 years of age)		
persons with disabilities		
frail individuals (with acute, chronic diseases, post-acute situations)		

How would you define your health status?
1) excellent
2) good
3) average
4) not good
5) very bad

In the past 12 months, have you been an active member of a political party, cultural association, or volunteer organization?
1) yes
2) no

How frequently do you go out with friends or acquaintances?
1) once or twice a week
2) once or twice a month
3) rarely
4) never

Indicate on a scale of 1 to 10 the degree of satisfaction with your social life, where 10 is maximum satisfaction and 1 is total dissatisfaction:
1 2 3 4 5 6 7 8 9 10

If he presented with fever, cough or other symptoms that could be traced to Covid-19:
1) I would worry immediately
2) I would wait a few days before getting worried
3) I would not think immediately at Covid-19.

If you thought you had symptoms attributable to Covid-19, who would you initially turn to? (1 answer only)
1) friends and relatives
2) primary care physician
3) emergency room
4) I would do an online search in order to better understand

If you have obvious symptoms attributable to Covid-19:
1) I would contact someone immediately
2) I would wait until the symptoms worsened before contacting someone

Do you own a smartphone?
1) yes, always connected
2) yes, but without a constant connection
3) no

Do you own a pc/tablet?
1) yes, always connected
2) yes, but without a constant connection
3) no

At home, your internet connection is:
1) stable
2) unstable

How would you rate your digital skills? Give a number from 1 to 10, where 10 indicates excellent digital skills and 1 none.
1 2 3 4 5 6 7 8 9 10

How would you rate the digital skills of your family members in general? Give a number from 1 to 10, where 10 indicates excellent digital skills and 1 none.
1 2 3 4 5 6 7 8 9 10

Do you use technological and digital means (e.g., apps, web, etc.):
1) always
2) often
3) a little
4) never

Do you find yourself not understanding the information on a website or app?
1) Always
2) Often
3) Sometimes
4) Rarely
5) Never

If this happens to you, do you find someone to help you? (skip only if you indicate "never" to the previous question)
1) Always
2) Often
3) Sometimes
4) Rarely
5) Never

Have you ever used telemedicine services?
1) Yes
2) No
3) Don't know

Have you ever used technological and digital means for:

	Yes	No
Booking appointments		
Prescription charges		
Services availability search		
Comparison of services quality		
Prescriptions renewal		
Entering data for self-assessment of my health		
Receiving results of tests performed		
Seeking advice from social health personnel		
Receiving post-therapeutic care		
Providing medical data to healthcare personnel		
Receiving information about medical treatments to follow		
Performing clinical monitoring		
Checking the outcome of therapy with the competent physicians		

Indicate whether you agree or disagree with the following statements:

	Completely disagree	Slightly agree	Strongly agree	Completely agree	Don't know
Telemedicine can help citizens manage their health more responsibly.					
Telemedicine can worsen the doctor-patient relationship.					
Telemedicine cannot have diagnostic purposes.					
Telemedicine can guarantee constant clinical monitoring.					
Telemedicine does not guarantee effective care.					
Thanks to Telemedicine, care can be more personalized.					
Telemedicine creates disparities between people with different income classes.					
Through Telemedicine, it can reach all citizens more efficiently, even in the most remote areas.					
Telemedicine puts citizens' data at risk.					
The development of Telemedicine will cause difficulties for the most vulnerable people (disabled, elderly, etc.).					
Telemedicine reduces the time for diagnosis and treatment.					
The more Telemedicine is developed in the Calabrian territory, the more the regional healthcare system will improve.					
